# Enhancing analytical performance of tyrosinase-based sensors with nanoparticles for detection of isoproterenol

**DOI:** 10.55730/1300-0527.3765

**Published:** 2025-09-04

**Authors:** Aysel OKTAY, Sevinç KURBANOĞLU, Gülsüm GÜNDOĞDU, Cem Bülent ÜSTÜNDAĞ, Frieder W. SCHELLER, Aysu YARMAN

**Affiliations:** 1 Department of Molecular Biotechnology, Faculty of Science, Turkish-German University, İstanbul, Turkiye; 2 Department of Bioengineering, Faculty of Chemical and Metallurgical Engineering, Yildiz Technical University, İstanbul, Turkiye; 3 Department of Analytical Chemistry, Faculty of Pharmacy, Ankara University, Ankara, Turkiye; 4 Department of Energy Science and Technology, Faculty of Science, Turkish-German University, İstanbul, Turkiye; 5 Health Biotechnology Joint Research and Application Center of Excellence, İstanbul, Turkiye; 6 Institute of Biochemistry and Biology, University of Potsdam, Potsdam, Germany

**Keywords:** Isoproterenol (isoprenaline), electrochemical biosensor, tyrosinase, iridium nanoparticles, poly(diallyldimethylammonium chloride)

## Abstract

In this work, electrochemical biosensors utilizing tyrosinase (Tyr) for the detection of the nonselective beta-adrenergic agonist isoproterenol (ISO) are presented. Three different configurations for immobilizing Tyr on a graphite electrode (GE) are compared: (1) GE modified with poly(diallyldimethylammonium chloride) (PDADMAC), PDADMAC/Tyr/GE; (2) PDADMAC combined with iridium nanoparticles (IrNPs) in a stepwise preparation, resulting in PDADMAC/IrNPs/Tyr/GE; and (3) a composite of PDADMAC and IrNPs mixed with Tyr at a 1:1 (v:v), forming PDADMAC/(IrNPs-Tyr)/GE. Surface morphology was characterized using scanning electron microscopy (SEM). Cyclic voltammetry (CV) and amperometry were applied to characterize the biosensor’s performance. Within the linear range of 5 μM to 211 μM, the biosensor PDADMAC/Tyr/GE exhibited a limit of detection (LOD) of 1.4 μM and a limit of quantification (LOQ) of 4.1 μM. PDADMAC/IrNPs/Tyr/GE displayed improved sensitivity with an LOD of 0.9 μM and an LOQ of 2.8 μM. The configuration PDADMAC/(IrNPs-Tyr)/GE demonstrated the best performance with an LOD of 0.3 μM and an LOQ of 0.8 μM. The slopes (0.0147 μA/M, 0.0096 μA/M, and 0.0031 μA/M for PDADMAC/(IrNPs-Tyr)/GE, PDADMAC/IrNPs/Tyr/GE, and PDADMAC/Tyr/GE, respectively) of the concentration dependencies for the three sensor modifications (which represent the analytical sensitivity) demonstrate the achieved enhancement of analytical performance by IrNPs. Furthermore, the biosensor’s ability to detect ISO in the presence of potential interferences, such as ascorbic acid, uric acid, and paracetamol, was assessed. Additionally, we demonstrated the biosensor’s potential to detect ISO in diluted spiked human serum samples.

## 1. Introduction

Isoproterenol (ISO), also known as isoprenaline, is a synthetic catecholamine drug with a phenolic structure that acts as a nonselective beta-adrenergic receptor agonist. It is primarily used as a bronchodilator for the treatment of chronic bronchitis, bronchial asthma, and emphysema. Additionally, it is used to manage certain heart-related conditions, such as bradycardia (slow heart rate) and heart block, due to its cardiac-stimulating effects. It exhibits effects such as increased cardiac muscle contraction, increased heart rate, and relaxation of bronchial smooth muscle [1–5]. Precise quantification of ISO becomes essential, necessitating the application of diverse analytical methodologies. Techniques such as spectroscopy [6], high-performance liquid chromatography (HPLC) [7], and capillary electrophoresis [8] have been widely used across various media. While these methods offer high sensitivity and selectivity, they often require expensive equipment, complex sample preparation, and lengthy analysis times [9,10]. In contrast, electrochemical enzymatic biosensors have emerged as promising and straightforward tools due to their distinct advantages, including high sensitivity, operational simplicity, cost-effectiveness, fast response times, and the potential for miniaturization and portability [10–12]. Among many enzymes, tyrosinase (Tyr, Enzyme Commission [EC] 1.14.18.1) also plays a role in drug sensing. Its catalytic activity includes the oxidation of catechol and catecholamines derived from dietary sources. Tyrosinase catalyzes two main reactions in the presence of molecular oxygen: the hydroxylation of monophenols to *o*-diphenols (monophenolase activity) and the oxidation of *o*-diphenols to *o*-quinones (diphenolase activity) (Equation 1). The resulting *o*-quinone can be analyzed by amperometry, measuring the cathodic reduction (Equation 2) [13–22].


(1) $$\text{Catechol}+1/2\;{\text{O}}_{2}\overset{\text{Tyrosinase}}{\to }O-\text{quinone}+{\text{H}}_{2}\text{O}$$

(2) $$O-\text{quinone}+2{\text{H}}^{+}+2{\text{e}}^{-}\overset{\text{Electrode}}{\to }\text{Catechol}$$

**Figure 1:**
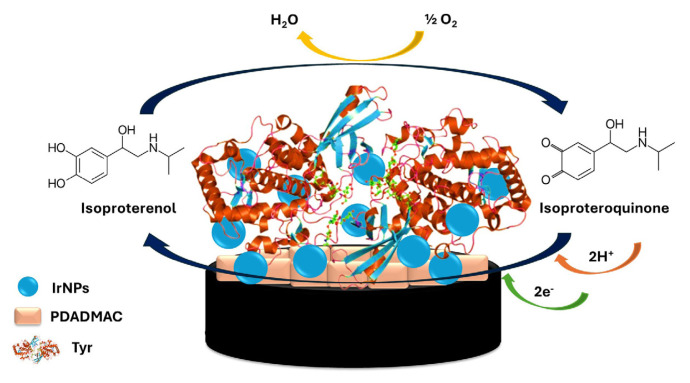
Schematic representation of the proposed biosensor, showing reactions involved in the ISO detection at the GE modified with PDADMAC and IrNPs.

Herein, we report for the first time a tyrosinase-based amperometric biosensor modified with iridium nanoparticles (IrNPs) for the detection of ISO (Figure 1). Tyrosinase oxidizes ISO to isoproteroquinone, which is reduced back at the electrode. The enzyme was immobilized on a graphite electrode (GE) modified with poly(diallyldimethylammonium chloride) (PDADMAC), which features a positively charged, hydrophilic quaternary ammonium group over a wide pH range. PDADMAC has been widely utilized for biomolecule immobilization in biosensor applications [23–26]. In this work, IrNPs were integrated to enhance sensitivity by increasing the electroactive surface area through a high surface area-to-volume ratio. Compared with other common nanomaterials, such as gold (Au) and silver (Ag) nanoparticles, IrNPs exhibit superior electrocatalytic activity, facilitating faster electron transfer and improving overall sensor performance. Additionally, IrNPs provide enhanced chemical stability and oxidation resistance, ensuring long-term operational reliability [27–29]. The biosensor was characterized through SEM, cyclic voltammetry (CV), and amperometry. Furthermore, the effect of interfering substances and measurement in diluted spiked human serum samples was studied.

## 2. Materials and methods

### 2.1. Chemicals and reagents

Tyrosinase from mushroom (EC 1.14.18.1, lyophilized powder, ≥1000 unit/mg), ISO hydrochloride (isoprenaline hydrochloride), PDADMAC (average Mw: 100,000–200,000 mol, 20 wt.% in H_2_O), paracetamol (PR; acetaminophen), uric acid (UA; 2,6,8-trihydroxypurine), ascorbic acid (AA), potassium phosphate dibasic and potassium phosphate monobasic were purchased from Sigma-Aldrich (St. Louis, MO, USA). Iridium nanoparticles with a mean diameter of 2 nm (0.05 mg/mL in citrate buffer) from DropSens (Oviedo, Spain) were used to modify the electrode surface. Human serum (Qualitrol) was purchased from Merck KGaA (Darmstadt, Germany). All solutions were prepared using ultrapure water from a New Human Power I water purification system. All reagents were of analytical grade and used without further purification.

### 2.2. Preparation of solutions

A 2-mM ISO solution was prepared in 50 mM phosphate buffer, pH 6.5, using a stock solution of 20 mM ISO (in 50 mM phosphate buffer, pH 6.5). Solutions of Tyr were subsequently prepared in 50 mM phosphate buffer (pH 6.5), with necessary dilutions performed using the same buffer.

### 2.3. Preparation of electrodes

GEs (3.05-mm diameter, effective surface area of 0.07 cm^2^ [30], counter flat top, purchased from Alfa Aesar, Ward Hill, MA, USA) were cleaned as described elsewhere [31]. The preparation of PDADMAC/IrNPs/Tyr-modified GEs involved three sequential steps. Initially, cleaned GEs were immersed in a PDADMAC solution (1:5 dilution with H_2_O) for 10 min at 4 °C using a dip-coating process. Following drying, 7 μL of ultrasonically dispersed IrNPs were applied to the PDADMAC-modified GEs and left to dry at room temperature. Subsequently, 7 μL of Tyr solution (10 mg/mL) was dropped onto the PDADMAC/IrNPs-modified GEs. For PDADMAC/(IrNPs-Tyr)-modified GEs, 7 μL of a 1:1 mixture (v:v) of Tyr and IrNPs was applied to the PDADMAC-modified electrodes. All modified electrodes were dried overnight at 4 °C and thoroughly rinsed with the measuring buffer.

### 2.4. Apparatus and electrochemical measurements

All electrochemical measurements were carried out in a homemade electrochemical cell with a volume of 2 mL employing a Palmsens potentiostat (Utrecht, The Netherlands). The experimental setup used a three-electrode configuration with a working electrode (GE), a platinum wire as the counter electrode, and an Ag/AgCl (3 M KCl) reference electrode.

Cyclic voltammograms were recorded in the presence of 5 μM ISO in 50 mM phosphate buffer at pH 6.5, scanning from −0.4 V to 1 V at 50 mV/s. Amperometric measurements were conducted in 50 mM phosphate buffer at pH 6.5 (stirring at 300 rpm at room temperature), applying a working potential of 0 V. Once baseline stabilization was achieved, the current was recorded following the addition of 2 mM ISO (in 50 mM phosphate buffer, pH 6.5) into the cell as a function of time.

The surface morphology of the electrodes was examined using a field-emission scanning electron microscope (Zeiss EVO 40, Merlin, Carl Zeiss, Oberkochen, Germany). Before imaging, the electrode samples were coated with a gold layer using the Emitech K550X system. Screen-printed carbon electrodes (SPCEs) comprising a 3 mm-diameter carbon working electrode, an Ag/AgCl reference electrode, and a carbon counter electrode were purchased from DropSens, Metrohm, Türkiye.

### 2.5. Interference analysis for ISO using PDADMAC/(IrNPs-Tyr)/GE and application in spiked human serum

In the presence of potential interferers, amperometry was employed to determine ISO using PDADMAC/(IrNPs-Tyr)/GE, maintaining a working potential of 0 V under stirring conditions at 300 rpm. Potential interfering substances, including 10 μM AA, 35 μM UA, and 10 μM PR, were introduced after attaining a stable current. Subsequently, 10 μM ISO was injected into the electrochemical cell. Additionally, recovery tests for ISO were conducted using synthetic human serum samples. A total of 163.5 μM ISO was added to the electrochemical cell containing human serum plasma diluted 1:10 with the buffer. Recovery results were evaluated using the calibration equations and reported as relative standard deviation (RSD%) values.

## 3. Results and discussion

### 3.1. Electrochemical characterization of the PDADMAC- and IrNPs-modified GEs

**Figure 2:**
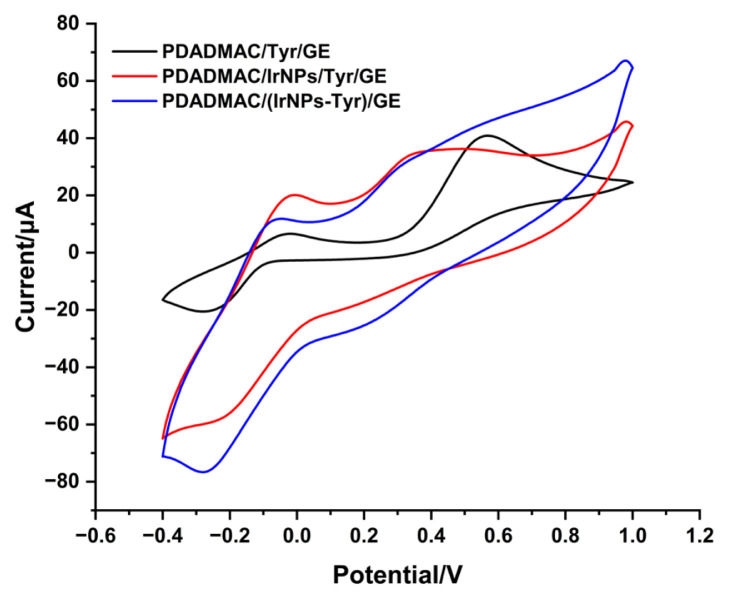
Cyclic voltammograms of PDADMAC/Tyr/GE, PDADMAC/IrNPs/Tyr/GE, and PDADMAC/(IrNPs-Tyr)/GE in the presence of 5 μM ISO in 50 mM phosphate buffer at pH 6.5 within a potential range of −0.4 V to 1 V at a scan rate of 50 mV/s.

To characterize the effect of the surface modifications on the ISO signal, CV was performed. The oxidation of ISO to the corresponding quinone and the corresponding back reaction are clearly visible in the potential range from 0 to −400 mV (Figure 2). In a forward scan, ISO, similar to other catecholamines, undergoes oxidation to form the corresponding quinone, isoproteroquinone (Figures 1 and 2). Then, in the reverse scan, the electrogenerated isoproteroquinone is reduced back to ISO [32–34]. Figure 2 clearly shows that IrNPs increase current signals. IrNPs and Tyr in PDADMAC/(IrNPs-Tyr)/GE biosensor resulted in the highest response to ISO. In our study, the synergistic effect of IrNPs and Tyr in the PDADMAC/(IrNPs-Tyr)/GE biosensor resulted in the greatest increase in response to ISO.

### 3.2. Influence of surface activity of tyrosinase on sensitivity

**Figure 3:**
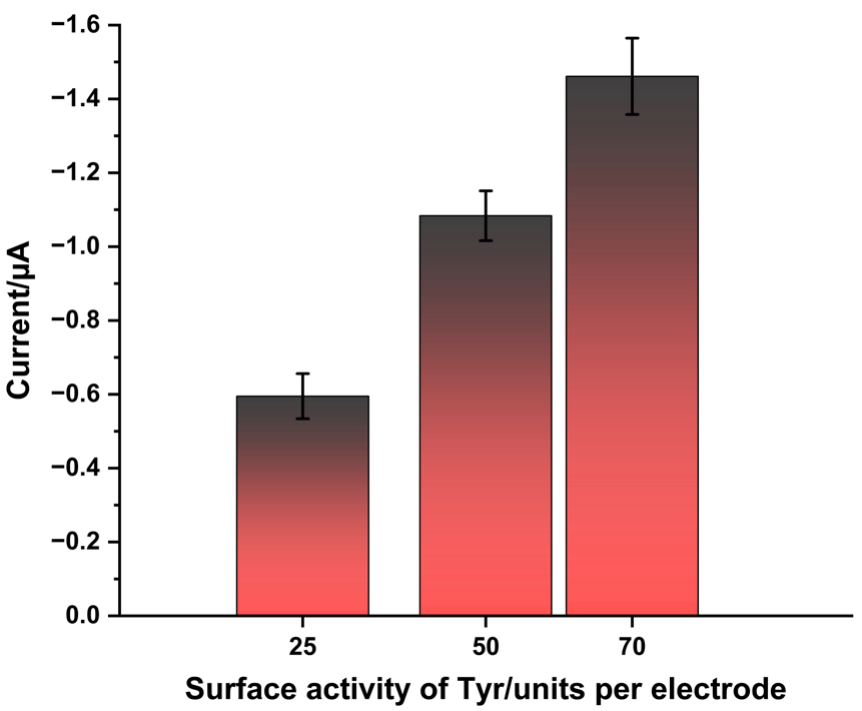
Influence of Tyr surface activity per electrode on sensitivity.

The influence of Tyr’s surface activity on sensitivity was characterized. The amount of Tyr immobilized on the working surface of the PDADMAC-modified electrodes ranged from 25 to 70 units per electrode (Figure 3). The increase in current change with the amount of immobilized enzyme shows that the degree of conversion of ISO increases with a higher surface concentration of the biocatalyst. This behavior indicates that the rate of the enzymatic reaction determines the sensor signal.

### 3.3. ISO detection using tyrosinase-based electrochemical sensors

**Figure 4:**
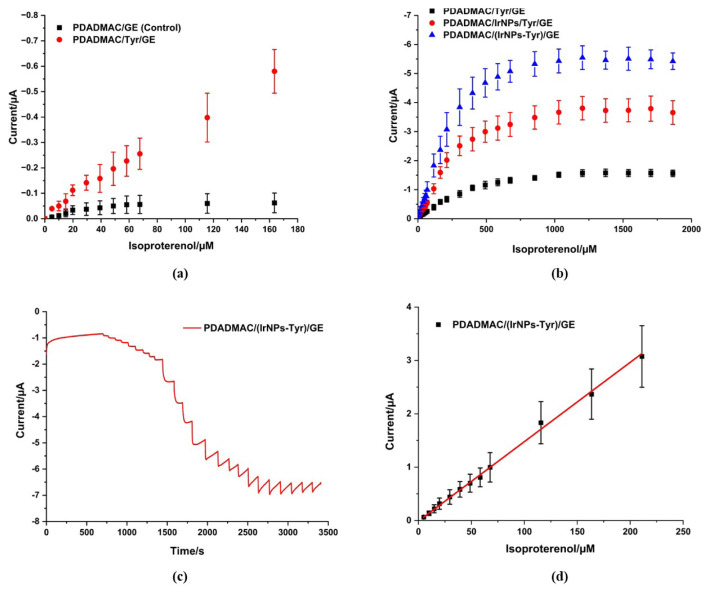
(a) Current–concentration dependencies of PDADMAC/GE as control and PDADMAC/Tyr/GE; (b) Current–concentration dependencies of PDADMAC/Tyr/GE, PDADMAC/IrNPs/Tyr/GE, and PDADMAC/(IrNPs-Tyr)/GE; (c) Amperometric responses of PDADMAC/(IrNPs-Tyr)/GE at 0 V on stepwise addition of ISO into 50 mM phosphate buffer at pH 6.5 during 300 rpm stirring conditions; (d) Analytical calibration of the PDADMAC/(IrNPs-Tyr)/GE.

Amperometry was performed to compare the different modifications of the GEs, modified with Tyr and IrNPs, for the detection of ISO. As shown in Figure 4a, the stepwise addition of ISO resulted in a negligible change in current at the PDADMAC-modified GE. In contrast, the immobilization of tyrosinase resulted in a cathodic current, attributed to the reduction of isoproteroquinone generated by the enzymatic reaction.

The limit of detection (LOD) and limit of quantification (LOQ) were determined based on the 3.3 s/m and 10 s/m principles, respectively, with “s” representing the standard deviation of the peak currents of the lowest analyte concentration and “m” representing the slope of the calibration curve [35]. In the linear range of 5 μM to 211 μM, PDADMAC/Tyr/GE exhibited LOD and LOQ values of 1.4 μM and 4.1 μM for ISO. The addition of IrNPs resulted in enhanced sensitivity, as evidenced by lower LOD and LOQ values for PDADMAC/IrNPs/Tyr/GE (LOD: 0.9 μM and LOQ: 2.8 μM) (Figure 4b).

IrNPs exhibit high electrocatalytic activity, which facilitates electron transfer between the electrode and electroactive species such as ISO and isoproteroquinone. This enhances the current response, enabling the detection of lower analyte concentrations. The increased surface area of IrNPs offers more active sites for both the enzyme and the electrochemical reaction. This behavior has been observed in previous studies [27–29,36,37]. Furthermore, the synergistic effect between IrNPs and Tyr improves electron mediation between the enzyme’s active site and the electrode, thereby contributing to lower LOD and LOQ values. Notably, even a 1:1 (v:v) dilution of Tyr with IrNPs did not result in a decrease in sensitivity. By contrast, PDADMAC/(IrNPs-Tyr)/GE displayed the highest performance with an LOD of 0.3 μM and an LOQ of 0.8 μM (Table 1).

**Figure 5:**
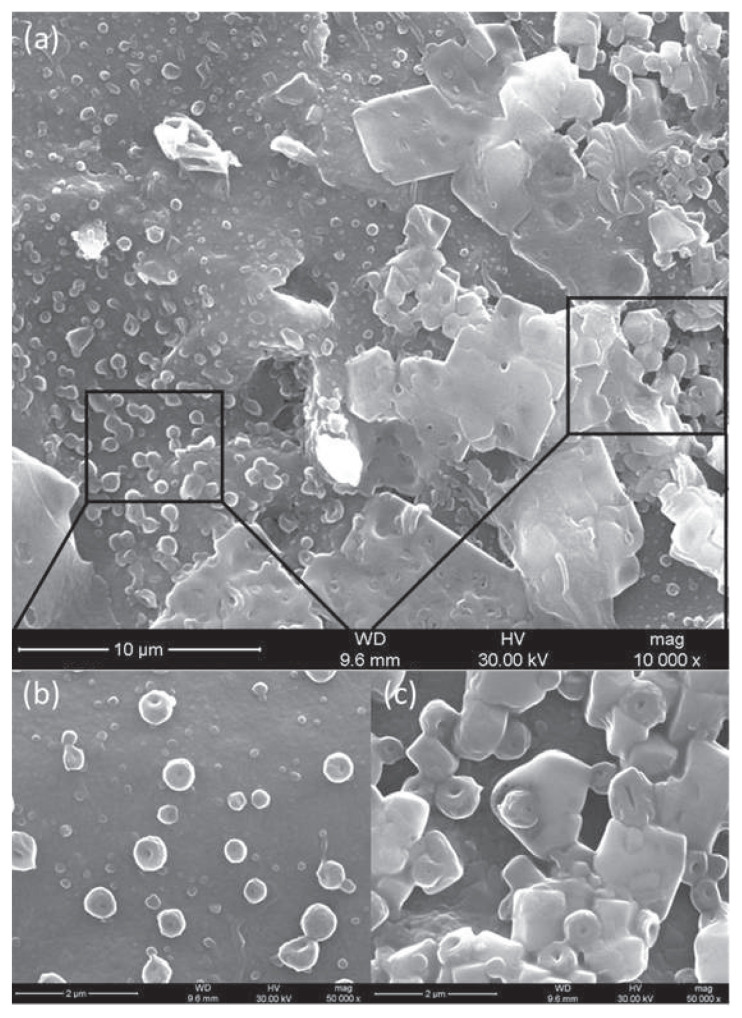
SEM images for PDADMAC/(IrNPs-Tyr)/SPCE. Scale bars: (a) 10 μm, (b) 2 μm, and (c) 2 μm.

The morphology of PDADMAC/(IrNPs-Tyr)/SPCE was characterized by SEM, as seen in Figure 5. Compared with the rough surface of the bare SPCE (Figure S1), Figure 5 shows the SEM images of low magnification (Figure 5a) and high magnification (Figures 5b and 5c) of PDADMAC/(IrNPs-Tyr)/SCPE, which reveal an aggregate-like structure. The spherical shape of the nanoparticles agglomerates of different sizes can be seen (Figure 5b). IrNPs were arranged on the surface (Figure 5). During the modification process of PDADMAC/(IrNPs-Tyr)/SPCE, particles formed denser flocs and larger aggregates on their surfaces, without uniformity, resulting in a significant increase in the effective electrode surface area for Tyr immobilization.

We compared the performance of our biosensor with other electrochemical sensors described in the literature and other analytical methods for detecting ISO (Tables S1 and S2). Our sensor yielded results comparable to those of the standard in terms of linear range and LOD.

### 3.4. Enzyme kinetics studies

Despite a nonsaturating concentration of the cosubstrate oxygen, the catalytic mechanism of the Tyr biosensor was modeled utilizing the Michaelis–Menten equation (for a single-substrate reaction) for PDADMAC/Tyr/GE, PDADMAC/IrNPs/Tyr/GE, and PDADMAC/(IrNPs-Tyr)/GE biosensors. Thus, this procedure provides “apparent values” for the Michaelis–Menten constant, K_m_. Initially, concentration dependencies for ISO were plotted (Figure 4b). Subsequently, a Lineweaver–Burk Plot was generated from the calibration curves by plotting 1/I (μA^−1^) vs. 1/[S] (μM^−1^). The Michaelis–Menten equation was used to calculate K_mapp_ and I_max_ values using the equation 1/I = 1/I_max_ + K_mapp_/I_max_ [S] [38,39]. These values are demonstrated in Table 2.

The biosensors’ kinetic behavior showed that the sequence of component immobilization strongly influences both K_mapp_ and I_max_. Among the three configurations tested, the PDADMAC/(IrNPs-Tyr)/GE system—where IrNPs and Tyr were coimmobilized—exhibited the lowest K_mapp_ and the highest I_max_ values. This arrangement may improve the enzyme’s accessibility to the substrate, resulting in a decrease in the apparent K_m_. In comparison, PDADMAC/IrNPs/Tyr/GE, where IrNPs were immobilized prior to Tyr, showed an improved current response compared with PDADMAC/Tyr/GE, owing to the increased surface activity of the enzyme induced by the IrNPs. However, despite the higher current response, the overall performance was lower than that of the coimmobilized system. The decreased K_mapp_ observed with IrNPs–Tyr coimmobilization may result from enhanced substrate–enzyme interaction, facilitated by the high surface area and electrocatalytic activity of IrNPs. The combination of enzymes and conductive nanoparticles in enzymatic biosensor studies can potentially influence K_mapp_ [40–44]. The K_mapp_ value indicates the substrate concentration at which the signal is half of the maximum. A lower K_mapp_ value suggests that the biosensor can detect the substrate at lower concentrations, potentially leading to lower detection limits. In comparison, a higher I_max_ value indicates that the biosensor generates a stronger electrical signal in the presence of ISO.

In summary, variations in K_mapp_ and I_max_ values suggest that modifications involving IrNPs—and particularly their coimmobilization strategy—enhance the biosensor’s performance for ISO detection.

### 3.5. Interference analysis for ISO using PDADMAC/(IrNPs-Tyr)/GE

The selectivity of the proposed PDADMAC/(IrNPs-Tyr)/GE biosensor was investigated by assessing potential interferences from AA, UA, and PR, based on their serum concentrations. The daily dosage of ISO is 0.2–10 mg, while the concentration in infusion solutions is typically 100 μM. These administrations result in a serum concentration of 1–10 μM [45].

The observed changes in the amperometric signal can be attributed either to direct electrode conversion or to enzymatic conversion into electroactive products. As shown in Figure S2, upon addition of AA and UA, which lack phenolic groups [46,47], there was no significant change in the amperometric signal because these substances were not electroactive at the applied potential. On the other hand, PR, which contains phenolic groups, produced notable cathodic currents due to conversion by Tyr [48,49]. However, ISO generated a higher cathodic current than PR. The activity of Tyr toward phenolic compounds could explain the observed variations in the amperometric signal, indicating that the biosensor signal for ISO in the presence of other Tyr substrates will be influenced.

### 3.6. Stability studies for PDADMAC/(IrNPs-Tyr)/GE

**Figure 6:**
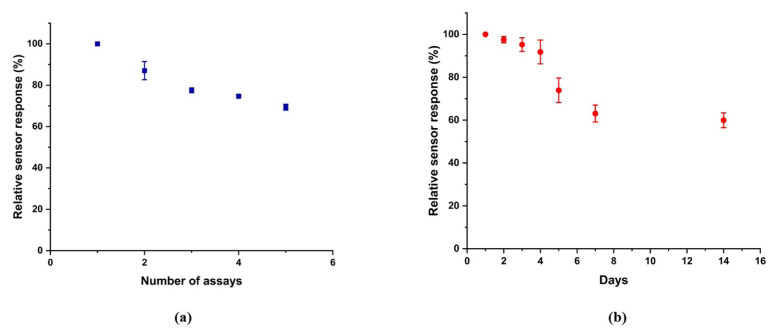
(a) Operational stability (b) Shelf life of the PDADMAC/(IrNPs-Tyr)/GE for 10 μM ISO at 300 rpm within a working potential of 0 V in 50 mM phosphate buffer pH 6.5.

The operational stability and shelf life of the developed biosensor were investigated (Figures 6a and 6b). For shelf-life studies, biosensors prepared for ISO detection were stored at 4 °C and monitored daily. While the biosensor retained approximately 97% of its initial current response to ISO after 1 day, this decreased to 63% of its original activity on the seventh day (Figure 6b). For operational stability, the biosensors were subjected to successive measurements of 10 μM ISO. The biosensor maintained 87% of its original activity after the first measurement (Figure 6a). The amperometric signal reached approximately 70% of the initial value after five consecutive measurements. This behavior reflects a decrease in the surface activity of Tyr, consistent with Figure 3, which shows that the sensor’s response is not yet under diffusion control.

### 3.7. Application in spiked human serum

To demonstrate the potential of the established biosensor, recovery tests were conducted by adding ISO to diluted synthetic human serum (1:10). Recovery percentages were calculated using Equation 3, and the results are shown in Table 3.


(3) $$\text{Recovery percentage}=\frac{{[\text{ISO}]}_{\text{Buffer}}}{{\left[\text{ISO}\right]}_{\text{Human serum}}}\times 100$$

The recovery percentage of 99.02% obtained from human serum samples for PDADMAC/(IrNPs-Tyr)/GE indicates minimal systematic error in the measurement. The RSD of 4.25% reflects the precision or uncertainty of the recovery value, indicating that the method provides reliable, consistent results.

We further evaluated the biosensor’s selectivity in diluted human serum in buffer (1:10) (Figure S3). The interference analysis yielded results consistent with those obtained in buffer solution alone. AA and UA did not significantly affect the amperometric signal. While PR produced a notable cathodic current, ISO generated a higher cathodic current, demonstrating the biosensor’s ability to detect ISO in a complex biological matrix.

## 4. Conclusions

In conclusion, we have developed amperometric biosensors utilizing PDADMAC, IrNPs, and Tyr for the detection of ISO. Notably, incorporating IrNPs into the biosensor has yielded the highest sensitivity. Specifically, the PDADMAC/(IrNPs-Tyr)/GE configuration demonstrates a broad linear range spanning from 5 μM to 211 μM, with an LOD of 0.3 μM and an LOQ of 0.8 μM for ISO detection. Furthermore, at the applied working potential, substances such as AA and UA, lacking phenolic groups, did not induce significant changes in the amperometric signal, as they are not electroactive; conversely, PR, containing phenolic groups, induced notable cathodic currents owing to its conversion by Tyr. Notably, the PDADMAC/(IrNPs-Tyr)/GE biosensor exhibits high sensitivity and the potential to detect ISO in diluted spiked human serum samples. This enhanced performance can be attributed to the presence of iridium nanoparticles, which facilitate electron transfer and further improve the biosensor’s sensitivity. Additionally, this biosensor configuration demonstrates the lowest K_mapp_ value, indicating its ability to detect ISO at lower concentrations. The biochemical design of this biosensor holds promise for analyzing other Tyr substrates, incorporating different enzymes, utilizing various conductive nanoparticles or polymers, and conducting enzymatic activity or inhibition studies on other drugs.

## Supplementary material

Figure S1 Bare screen-printed electrode (SPCE).

Figure S2 Effect of potentially interfering substances, including 10 μM AA, 35 μM UA, and 10 μM PR, on the PDADMAC/(IrNPs-Tyr)/GE on 10 μM ISO at 300 rpm within a working potential of 0 V in 50 mM phosphate buffer pH 6.5.

Figure S3 Effect of potentially interfering substances, including 10 μM AA, 35 UA, and 10 μM PR, on the PDADMAC/(IrNPs-Tyr)/GE on 10 μM ISO at 300 rpm within a working potential of 0 V in human serum (1:10 dilution).

**Table S1:** Comparison of some selected electrochemical detection platforms for ISO.

Sensor	Method	Linear range	LOD	Ref
CB/GO/CuNPs PEDOT:PSS/GCE	SWV	8–50 μM	1.9 μM	[ 1]
DMD-AuNPs/GCE	DPV	0.5–800 μM	0.21 μM	[ 2]
GQDs/SPE	DPV	1–900 μM	0.6 μM	[ 3]
MoS _2_ NSs/GSPE	CA	0.07–550 μM	0.03 μM	[ 4]
HNCS/GCE	LSV	0.2–2 μM	60 nM	[ 5]
		2–30 μM		
3,4′-AAGPE	CA	0.025–20 μM	12 nM	[ 6]
DHB/CNT	DPV	10–6000 μM	1.24 μM	[ 7]
PGRMMWCNTPE	CA	0.8–570 μM	0.47 μM	[ 8]
FMAMCNTPE	CA	0.5 – 50 μM	0.2 μM	[ 9]
CuHCF/CPE	CV	196–1070 μM	80 μM	[ 10]
La ^3+/^ZnO NFs/NFMF2A/CPE	DPV	0.1–400 μM	0.05 μM	[ 11]
MCO/GCE	DPV	0.2–121 μM	0.005 μM	[ 12]
BNHC/GCE	SWV	0.05 – 15 μM	6.8 nM	[ 13]
		15 – 70 μM		
MWC/ZnCo-ZIF/IL/CPE	DPV	0.04–580 μM	9.8 nM	[ 14]
PDADMAC/Tyr/GE	Amperometry	5–211 μM	1.4 μM	This study
PDADMAC/IrNPs/Tyr/GE	Amperometry	5–211 μM	0.9 μM	This study
PDADMAC/(IrNPs-Tyr)/GE	Amperometry	5–211 μM	0.3 μM	This study

Abbreviations: CB: carbon black; GO: graphene oxide; CuNPs: copper nanoparticles; PE–DOT:PSS: poly(3,4-ethylenedioxythiophene)-poly(styrenesulfonate); GCE: glassy carbon electrode, SWV: square wave voltammetry; DMD: 5-(1,3-dithiolane-2-eyl)-3-methyl benzene-1,2-diol; AuNPs: gold nanoparticles; DPV: differential pulse voltammetry; GQDs: graphene quantum dots; SPE: screen-printed electrode; MoS_2_ NSs: molybdenum disulfide nanosheets; GSPE: graphite screen-printed electrode; CA: chronoamperometry; HNCS: hollow nitrogen-doped carbon spheres; LSV: linear sweep voltammetry; 3,4′-AA: 3-(4′-amino-3′-hydroxy-biphenyl-4-yl)-acrylic acid; GCPE: graphene oxide nanosheets paste electrode; DHB: 2-((7-(2,5-dihydrobenzylideneamino)heptylimino)methyl) benzene-1,4-diol; CNT: carbon nanotubes; PGRMMWCNTPE: pyrogallol red modified-multiwalled carbon nanotube paste electrode; FMAMCNTPE: ferrocenemonocarboxylic acid modified carbon nanotubes paste electrode; CuHCF: copper (II) hexacyanoferrate (III); CPE: carbon paste electrode; NFs: nanoflowers; NFMF2A: N-(ferrocenylmethylidene)fluoren-2-amine; MCO: manganese cobalt oxide; BNHC: bowl-shaped N-doped hollow carbon sphere-containing mesoporous nanomaterials; MWC: multiwalled carbon nanotubes; ZnCo-ZIF: ZnCo-zeolitic-imidazolate framework; IL: ionic liquid.

**Table S2:** Comparison of other detection platforms for ISO.

Method	Detection	Linear range	LOD	Ref
HPLC	Electrochemical	from basal concentrations to 1.62 μM	<8.07×10 ^−6^ μM	[ 15]
Spectrophotometry	Flow-injection	123–738 μM	62.5 μM	[ 16]
Spectrophotometry	Automated flow-injection	40.4–1211 μM	4.8 μM	[ 17]
Spectrophotometry	Flow-injection	20–200 μM	0.8 μM	[ 18]
Capillary electrophoresis	Indirect electrochemiluminescence	0.2–50 μM	0.084 μM	[ 19]
Chemiluminescence	-	0.94–236 μM	0.236 μM	[ 20]
Chemiluminescence	Flow-injection	10–100 ng mL ^−1^	5 ng mL ^−1^	[ 21]
Amperometry	PDADMAC/Tyr/GE	5–211 μM	1.4 μM	This study
Amperometry	PDADMAC/IrNPs/Tyr/GE	5–211 μM	0.9 μM	This study
Amperometry	PDADMAC/(IrNPs-Tyr)/GE	5–211 μM	0.3 μM	This study

References1

WongA
SantosAM
SilvaTA
Fatibello-FilhoO

Simultaneous determination of isoproterenol, acetaminophen, folic acid, propranolol and caffeine using a sensor platform based on carbon black, graphene oxide, copper nanoparticles and PEDOT:PSS
Talanta
2018
183
329
338
10.1016/j.talanta.2018.02.066
29567183
2

Mazloum-ArdakaniM
DehghaniZ
KhoshrooA

Self-assembled monolayers of organosulfur derivative on gold nanoparticles as electrochemical sensor for determination of isoprenaline
Journal of the Iranian Chemical Society
2018
15
1061
1068
10.1007/s13738-018-1303-5
3

DourandishZ
BeitollahiH

Electrochemical sensing of isoproterenol using graphite screen-printed electrode modified with graphene quantum dots
Analytical and Bioanalytical Electrochemistry
2018
10
192
202
4

BaezzatMR
TavakkoliN
ZamaniH

Electrochemical sensing platform based on modified graphite screen-printed electrode to determine isoproterenol in the presence of theophylline and acetaminophen
Journal of Materials Science: Materials in Electronics
2022
33
1173
1182
10.1007/s10854-021-07399-9
5

ShahrokhianS
PanahiS
SalimianR

An electrochemical sensing platform based on nitrogen-doped hollow carbon spheres for sensitive and selective isoprenaline detection
Journal of Electroanalytical Chemistry
2019
847
113196
10.1016/j.jelechem.2019.113196
6

MohammadiSZ
BeitollahiH
FadaeianH

Voltammetric determination of isoproterenol using a graphene oxide nanosheets paste electrode
Journal of Analytical Chemistry
2018
73
705
712
10.1134/S1061934818070122
7

Mazloum-ArdakaniM
SabaghianF
KhoshrooA
NaeimiH

Simultaneous determination of the concentrations of isoproterenol, uric acid, and folic acid in solution using a novel nanostructure-based electrochemical sensor
Chinese Journal of Catalysis
2014
35
4
565
572
10.1016/s1872-2067(14)60027-9
8

KeyvanfardM
AlizadK

Determination of isoproterenol in pharmaceutical and biological samples using a pyrogallol red multiwalled carbon nanotube paste electrode as a sensor
Cuihua Xuebao/Chinese Journal of Catalysis
2016
37
4
579
583
10.1016/S1872-2067(15)61036-1
9

EnsafiAA
MalehHK

A multiwall carbon nanotubes paste electrode as a sensor and ferrocenemonocarboxylic acid as a mediator for electrocatalytic determination of isoproterenol
International Journal of Electrochemical Science
2010
5
10
1484
1495
10

BonifácioVG
MarcolinoLH
TeixeiraMFS
Fatibello-FilhoO

Voltammetric determination of isoprenaline in pharmaceutical preparations using a copper(II) hexacyanoferrate(III) modified carbon paste electrode
Microchemical Journal
2004
78
1
55
59
10.1016/j.microc.2004.03.010
11

PourtaheriE
TaherMA
BeitollahiH
HosseinzadehR

Co-detection of isoprenaline and paracetamol in biological and pharmaceutical media by a feather-like La3 + /ZnO nanoflowers and N-(ferrocenylmethylidene) fluoren-2-amine-modified carbon paste electrode: analysis of a novel sensor
Journal of the Iranian Chemical Society
2020
17
1447
1456
10.1007/s13738-020-01870-w
12

ManjulaN
ChenSM

Electrochemical sensors for β-adrenoceptor agonist isoprenaline analysis in human urine and serum samples using manganese cobalt oxide-modified glassy carbon electrode
New Journal of Chemistry
2021
45
20
9084
9095
10.1039/D1NJ01009C
13

ZhouM
SuG
PuS
TangT
LuD


Bowl-shaped hollow N-doped carbon as an electrochemical platform for the highly selective and sensitive electrochemical detection of isoprenaline in pharmaceutical injection
Electroanalysis
2023
35
7
e202200499
10.1002/elan.202200499
14

MohammadiSZ
TajikS
BadriY
TezerjiLS
MousazadehF


Sensitive determination of the cardiac drug isoproterenol in the presence of acetaminophen using modified electrode with multiwall carbon nanotube/ZnCo-Zeolite imidazole frameworks and ionic liquid
Diamond and Related Materials
2024
148
111445
10.1016/j.diamond.2024.111445
15

WangY
FiceDS
YeungPKF

A simple high-performance liquid chromatography assay for simultaneous determination of plasma norepinephrine, epinephrine, dopamine and 3,4-dihydroxyphenyl acetic acid
Journal of Pharmaceutical and Biomedical Analysis
1999
21
3
519
525
10.1016/S0731-7085(99)00117-X
10701418
16

LupettiKO
VieiraIC
Fatibello-FilhoO

Flow injection spectrophotometric determination of isoproterenol using an avocado (Persea americana) crude extract immobilized on a controlled-pore silica reactor
Talanta
2002
57
1
135
143
10.1016/S0039-9140(01)00681-6
18968613
17

SolichP
PolydorouCK
KoupparisMA
EfstathiouCE

Automated flow-injection spectrophotometric determination of catecholamines (epinephrine and isoproterenol) in pharmaceutical formulations based on ferrous complex formation
Journal of Pharmaceutical and Biomedical Analysis
2000
22
5
781
789
10.1016/S0731-7085(00)00291-0
10815721
18

NevadoJJB
GallegoJML
LagunaPB

Spectrophotometric determination of catecholamines with metaperiodate by flow-injection analysis
Analytica Chimica Acta
1995
300
1–3
293
297.v
19

LiuYM
CaoJT
ZhengYL
ChenYH

Sensitive determination of norepinephrine, synephrine, and isoproterenol by capillary electrophoresis with indirect electrochemiluminescence detection
Journal of Separation Science
2008
31
13
2463
2469
10.1002/jssc.200800034
18646273
20

ZhouGJ
ZhangGF
ChenHY

Development of integrated chemiluminescence flow sensor for the determination of adrenaline and isoprenaline
Analytica Chimica Acta
2002
463
2
257
263
10.1016/S0003-2670(02)00418-X
21

ZhangG
TangY
ShangJ
WangZ
YuH


Flow-injection chemiluminescence method to detect a β2 adrenergic agonist
Luminescence
2015
30
1
102
109
10.1002/bio.2698
24830367


## Figures and Tables

**Table 1:** Regression data for detection of ISO using PDADMAC/Tyr/GE, PDADMAC/IrNPs/Tyr/GE, and PDADMAC/(IrNPs-Tyr)/GE biosensors.

Regression data	PDADMAC/Tyr/GE	PDADMAC/IrNPs/Tyr/GE	PDADMAC/(IrNPs-Tyr)/GE
Linear range (μM)	5–211	5–211	5–211
Slope (μA.M^−1^)	0.0031	0.0096	0.0147
Intercept (μA)	0.0364	−0.0325	0.0027
Standard error of slope	0.78 × 10^−4^	1.88 × 10^−4^	2.21 × 10^−4^
Standard error of intercept	0.007	0.017	0.020
Correlation coefficient	0.9939	0.9962	0.9977
Limit of detection (μM)	1.4	0.9	0.3
Limit of quantification (μM)	4.1	2.8	0.8

**Table 2:** K_mapp_ and I_max_ values calculated using the Michaelis–Menten model.

Biosensor	K_mapp_ (μM)	I_max_ (μA)
PDADMAC/Tyr/GE	385.96	2.00
PDADMAC/IrNPs/Tyr/GE	309.96	4.62
PDADMAC/(IrNPs-Tyr)/GE	266.98	6.69

**Table 3:** Detection of ISO from synthetic human serum (1:10 dilution).

Parameters	Serum containing ISO
Amount added (μM)	163.5
Found (μM)	161.9
Average recovered (%)	99.02
RSD% of recovery	4.25
Bias%	−1.14
