# Supplementary figures and images for: Enhancing analytical performance of tyrosinase-based sensors with nanoparticles for detection of isoproterenol
Source: Turk J Chem. 2025 Sep 4;49(6):706–16. doi: 10.55730/1300-0527.3765 (PMC12779017; doi:10.55730/1300-0527.3765)

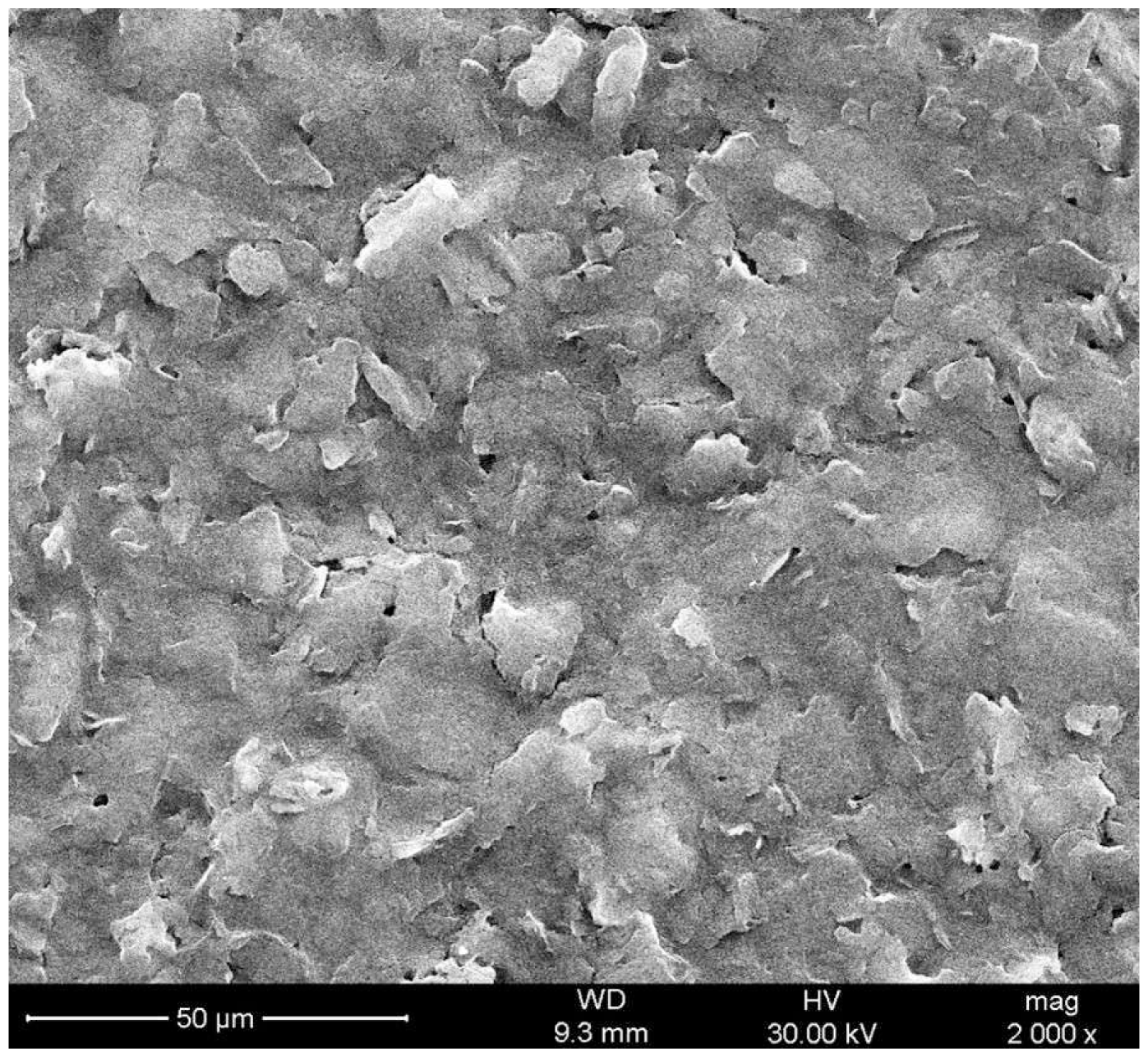

Supplement: Figure S1 — Bare screen-printed electrode (SPCE). [file tjc-49-06-706s1.tif]

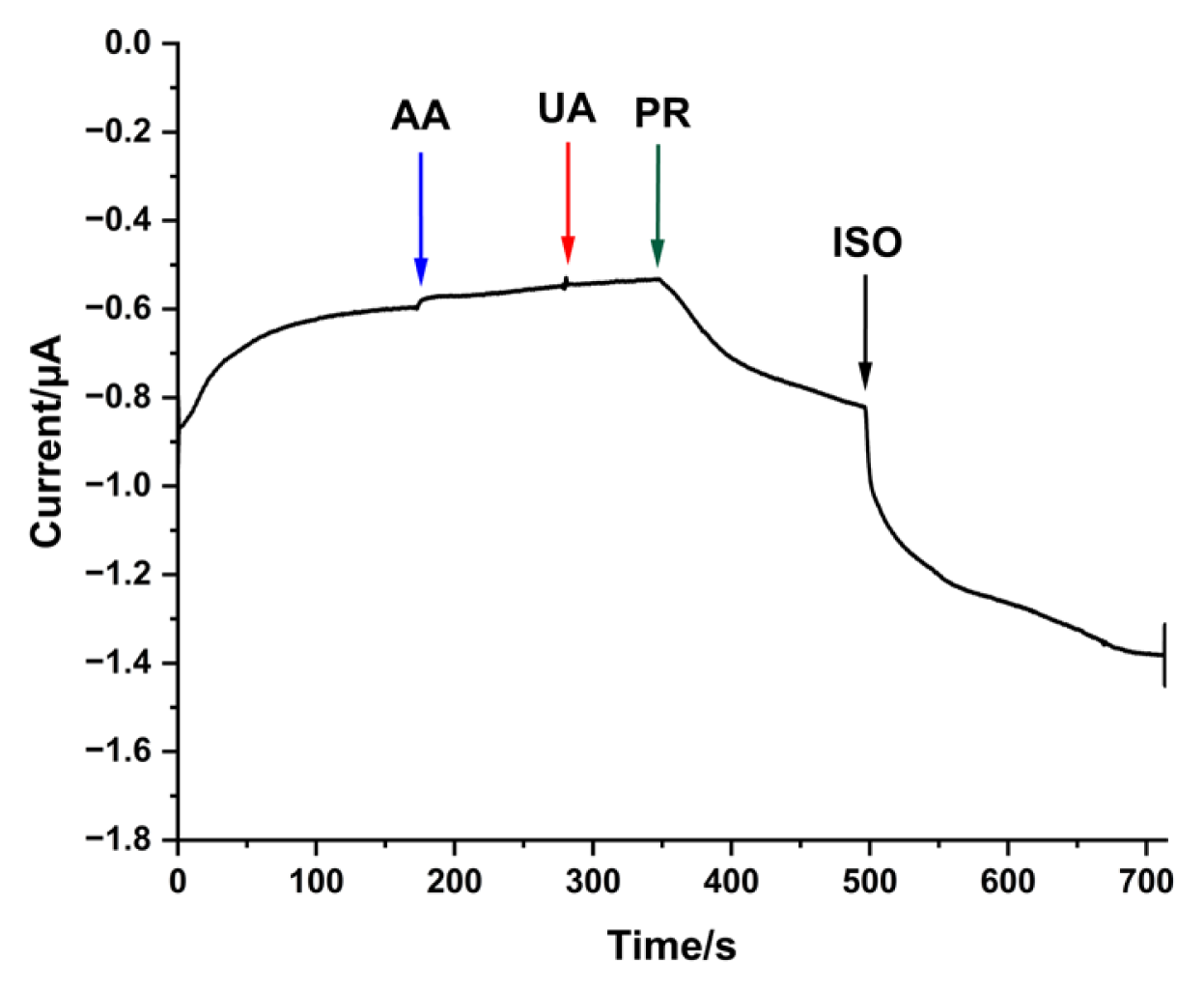

Supplement: Figure S2 — Effect of potentially interfering substances, including 10 μM AA, 35 μM UA, and 10 μM PR, on the PDADMAC/(IrNPs-Tyr)/GE on 10 μM ISO at 300 rpm within a working potential of 0 V in 50 mM phosphate buffer pH 6.5. [file tjc-49-06-706s2.tif]

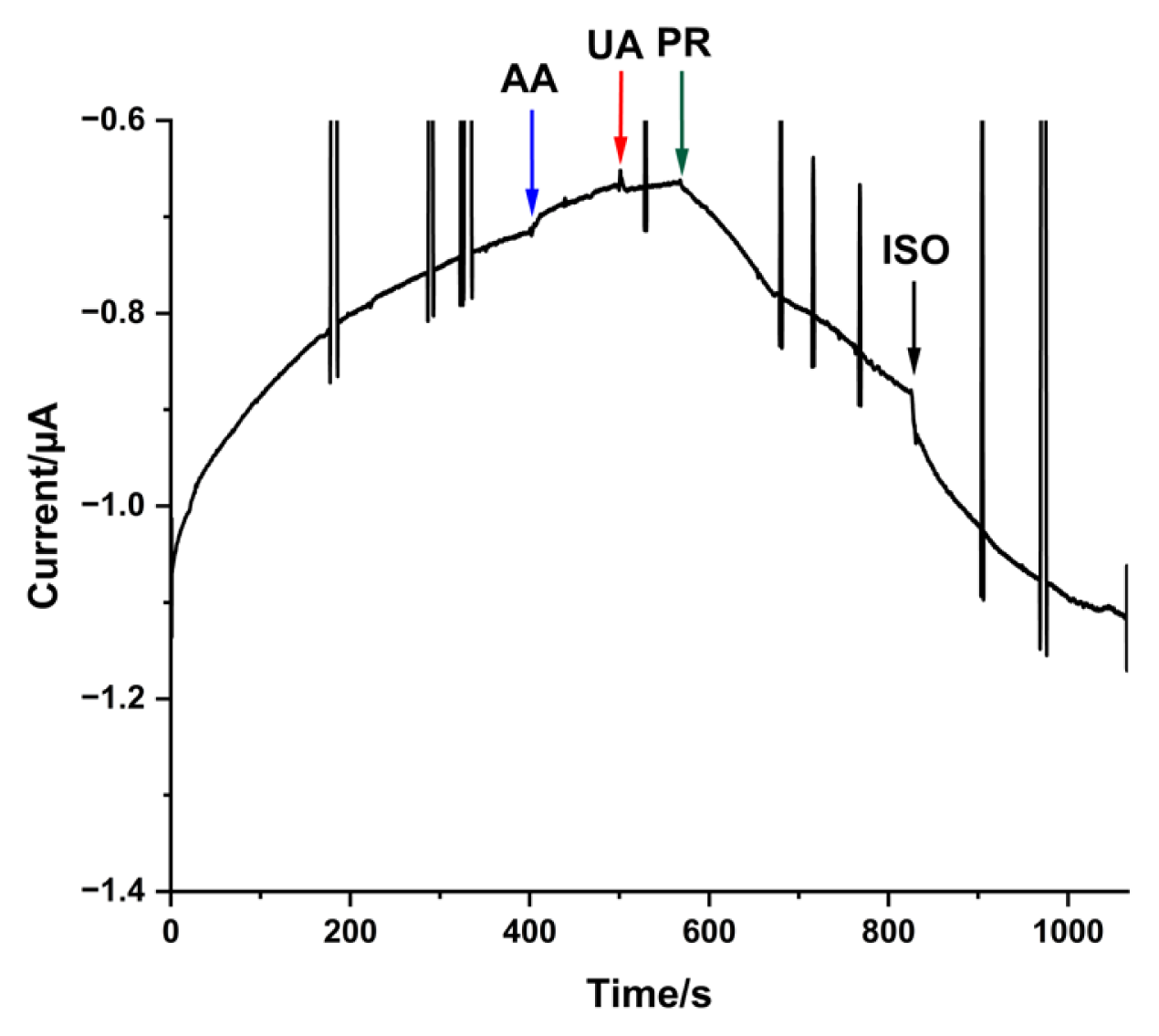

Supplement: Figure S3 — Effect of potentially interfering substances, including 10 μM AA, 35 UA, and 10 μM PR, on the PDADMAC/(IrNPs-Tyr)/GE on 10 μM ISO at 300 rpm within a working potential of 0 V in human serum (1:10 dilution). [file tjc-49-06-706s3.tif]
